# The long term effect of metabolic profile and microbiota status in early gastric cancer patients after subtotal gastrectomy

**DOI:** 10.1371/journal.pone.0206930

**Published:** 2018-11-05

**Authors:** Xi-Hsuan Lin, Kuo-Hung Huang, Wei-Hung Chuang, Jiing-Chyuan Luo, Chung-Chi Lin, Po-Hsiang Ting, Shih-Hao Young, Wen-Liang Fang, Ming-Chih Hou, Fa-Yauh Lee

**Affiliations:** 1 Department of Medicine, School of Medicine, National Yang Ming University, Taipei, Taiwan; 2 Division of Gastroenterology and Hepatology, Department of Medicine, Taipei Veterans General Hospital, Taipei, Taiwan; 3 Department of Surgery, School of Medicine, National Yang Ming University, Taipei, Taiwan; 4 Division of General Surgery, Department of Surgery, Taipei Veterans General Hospital, Taipei, Taiwan; 5 Institute of Biomedical Informatics, National Yang Ming University, Taipei, Taiwan; 6 Healthcare and Management Center, Taipei Veterans General Hospital, Taipei, Taiwan; West China Hospital, Sichuan University, CHINA

## Abstract

Long term effects of subtotal gastrectomy on gut microbiota modifications with subsequent metabolic profiles are limited. We aimed to investigate and compare long-term effects of metabolic profiles and microbiota status in early gastric cancer patients post curative subtotal gastrectomy to the controls. In this cross-sectional study, we analyzed type II diabetes mellitus and metabolic syndrome occurrence in two groups: 111 patients after curative subtotal gastrectomy with Billroth II (BII) anastomosis and Roux-en-Y gastrojejuno (RYGJ) anastomosis and 344 age-sex matched controls. Fecal samples from those with BII, RYGJ, and controls were analyzed by next-generation sequencing method. Metabolic syndrome and type II diabetes mellitus occurrences were significantly lower in patients after subtotal gastrectomy with RYGJ than in controls over the long term (> 8 years) follow-up (P < 0.05). The richness and diversity of gut microbiota significantly increased after subtotal gastrectomy with RYGJ (P < 0.05). Compared with the control group, the principal component analysis revealed significant differences in bacterial genera abundance after subtotal gastrectomy with BII and RYGJ (P < 0.001). Genera of *Oscillospira*, *Prevotella*, *Coprococcus*, *Veillonella*, *Clostridium*, *Desulfovibrio*, *Anaerosinus*, *Slackia*, *Oxalobacter*, *Victivallis*, *Butyrivibrio*, *Sporobacter*, and *Campylobacter* shared more abundant roles both in the RYGJ group and BII groups. Early gastric cancer patients after subtotal gastrectomy with RYGJ had a lower occurrence of metabolic syndrome and type II diabetes mellitus than the controls during long term follow-up. In parallel with the metabolic improvements, gut microbial richness and diversity also significantly increased after subtotal gastrectomy with RYGJ.

## Introduction

Gastric cancer is currently the fourth most common malignancy and the second leading cause of cancer death in the world [1]. Nearly half of global gastric cancer cases occur in Eastern Asia.^1^ Metabolic syndrome (MS), characterized by a range of metabolic disorders, including abdominal obesity, insulin resistance/glucose intolerance, atherogenic dyslipidemia, raised blood pressure, and proinflammatory and prothrombotic states, is increasing in Asia [2]. Recent studies also have demonstrated a carcinogenic function for MS in many types of cancer, including gastric cancer [3–5].

Previous studies have revealed that gastrectomy with duodenal bypass caused higher diabetes mellitus (DM) remission in gastric cancer patients [6–9]. Furthermore, gastric bypass surgery has also been reported to have a higher remission rate of MS than that of purely restrictive procedures in bariatric surgery [10, 11]. The underlying mechanism for type II DM and MS remission after gastric bypass surgery is interesting and still undergoing investigation. Although the purposes of bariatric and gastric cancer surgeries are completely different, there are some anatomical and technical similarities between the two procedures. Recent work has indicated that the gut microbiota may mediate some of the beneficial effects of bariatric surgery; changes in the composition and diversity of the gut microbiota have been observed in humans after short term bariatric bypass surgery [12–14]. However, the long term effects on the metabolic profiles and microbiota status after subtotal gastrectomy due to curable gastric cancer is interesting and waiting to be understood.

In line with this thinking, we investigated and compared the long term effects of metabolic profiles and microbiota statuses in early gastric cancer patients who underwent subtotal gastrectomy and those of controls without any gastrointestinal (GI) tract surgery.

## Materials and methods

### Study population

We performed a cross-sectional study at Taipei Veterans General Hospital, 111 subjects at outpatient clinics who had early gastric cancer and underwent curative subtotal gastrectomy for primary gastric cancer in Taipei Veterans General Hospital between 2001 and 2007 were identified and enrolled successively in our study group. The time of follow-up after surgery (BII or RYGJ) to study enrollment was 8.25 years (ranging 6.50 to 10.25 years). Early gastric cancer is defined by the Japanese Research Society for Gastric Cancer as cancer in which the tumor cells invade only the mucosal and submucosal layers [15]. Subtotal gastrectomy was defined as the surgical removal of the distal two thirds of the stomach. The restorative methods for digestive continuity after subtotal gastrectomy were as follows: 1.) Billroth II anastomosis (BII) and 2.) Roux-en-Y gastrojejuno anastomosis (RYGJ). Patients with the several conditions were excluded: 1.) aged < 20 years; 2.) other underlying malignancies; 3.) pre- and postoperative chemotherapy or chemoradiotherapy for gastric cancer; 4.) other endocrine disorders such as DM, thyroid, pituitary or adrenal disease; 5.) moderate to severe cardiovascular, pulmonary, hepatic, or renal disease; 6.) recurrent or uncured gastric cancer even after curative gastric surgery; 7) the occurrence of complications after gastric cancer resection including anastomotic leakage, bleeding, intermittent intestinal adhension, dumping syndrome, etc. In addition, patients who had received proton pump inhibitors, histamine-2 receptor antagonists, nonsteroidal anti-inflammatory drugs, antibiotics, or probiotics within one month of sample collection were excluded. In another part, age-sex, time of follow-up matched subjects without GI tract surgery were also enrolled as the control group with the same exclusion criteria as study group. Three-hundred forty-four subjects were enrolled as the controls. This study complies with the standards of the Declaration of Helsinki and current ethical guidelines and has been approved by the Institutional Review Board (IRB) of Taipei Veterans General Hospital, Taiwan, dated on July, 15, 2016 with the number of 2016-07-008B.

### Anthropometric and laboratory measurement

After signing informed consents, detailed clinical and laboratory data, including the presence of type II DM and dyslipidemia were measured and recorded at out-patient clinical departments. Anthropometric measurements (such as body height and weight, waist circumference, body mass index [BMI], and blood pressure [BP]) were taken by an experienced nursing staff. Blood tests including serum glucose, total cholesterol levels, high-density lipoprotein (HDL), low-density lipoprotein (LDL)-cholesterol, triglycerides) were collected after an overnight fast. MS was measured and diagnosed if three or more criteria were met: 1.) abdominal obesity, waist circumference 90 cm in males and ≥80 cm in females; 2.) high blood pressure, ≥130 mmHg systolic, ≥85 mmHg diastolic, or current medication for hypertension; 3.) high serum fasting glucose, ≥100 mg/dL or current use of anti-diabetic therapy; 4.) low high-density lipoprotein (HDL)-cholesterol <40 mg/dL in males and <50mg/dL in females; and 5.) hypertriglyceridemia ≥150mg/dL [16].

### Stool bacterial genomic DNA extraction and PCR amplification

We further analyzed anthropometric, laboratory, and fecal microbiome from 14 patients who had undergone subtotal gastrectomy with BII, 14 study patients with RYGJ, and 14 controls of the same study subjects. Fresh stool samples were collected at home at the same day or one day before blood measurement and immediately frozen in their home freezers at −20°C which were then delivered to the hospital within four hours in insulating polystyrene foam containers and stored at −80°C until DNA extraction. Overall, the mean time interval between surgery and measurement was 8.25 years.

Bacterial genomic microbial DNAs were extracted using the QIAamp DNA Stool Mini Kit (Qiagene, MD, USA) according to the manufacturer’s protocols. Briefly, tissue samples (180~220 mg) yielded 5–100 μg genomic DNA for direct use in 16S rRNA gene sequencing. The amounts and quality of isolated genomic DNA were determined with NanoDrop ND-1000 (Thermo Scientific, Wilmington, DE, USA). Prior to 16S rRNA sequencing, genomic DNA was stored at –80°C for further analysis. One microliter of sample DNA (10pg~500 ng) was used as template in a PCR reaction for bacteria 16S rRNA hyper-variable region V3–V4. The primer set for the reaction was chosen with 341F_V3_illumina (5′ - CCTACGGGNGGCWGCAG-3′) and 805R_V4_illumina (5′ - GACTACHVGGGTATCTAATCC -3′) [17]. PCR consisted of an initial denaturation at 94°C for 2 min, 30 cycles of 92°C for 20 sec, 55°C for 30 sec and 68°C for 1 min for amplification, 68°C for 1 min to finish replication on all templates, and stored at 4°C. Dual-indexes (barcodes) were used for each sample before sequencing and next-generation sequencing was performed by the Illumina MiSeq Desktop Sequencer following the standard protocol.

### Data processing and statistical analysis

From the initial clean reads, several steps were performed to obtain the final effective reads: 1.) demultiplex each sample by dual-index using “in-house script”; 2.) join paired-end (PE) reads using program “PEAR”; 3.) trim primer sequences from joined reads using program “AlienTrimmer”; 4.) trim off low quality end sequences by sliding windows (5 nt) with average quality value under 10 and screen out short sequences less than 200 nt using program “Trimmomatic”; and 5.) filter out chimeric reads using software package “Mothur” v.1.33.3 (Department of Microbiology & Immunology at The University of Michigan, USA). 16S rDNA analysis with the Greengenes 16S rRNA Taxonomy Database (gg_13_8) was performed by the software package ‘Mothur’ version 1.33.3 and ‘QIIME’ version 1.80 [18]. We calculated the abundance-based coverage estimator (ACE), Chao richness estimator, and the Shannon diversity index (SI) using the Mothur program.

All statistical analyses of bacterial community were performed using R software (http://www.r-project.org/), unless otherwise specified. Gene copy number-corrected abundance of genera was total-sum scaled per sample [19].

Taxonomic microbiota profiles were submitted to principal component analysis (PCA) which was performed on log-transformed data using the R package ADE4 to analyze genera abundance between groups [20].Between-group inertia percentages was tested (Monte-Carlo test with 10000 permutations) to determine the *P* values of the PCA results. Statistically significant differences in the relative abundance of taxa associated with groups of patients were performed using linear discriminant analysis (LDA) effect size (LEfSe) with α = 0.05 (Kruskal-Wallis and Wilcoxon tests) and effect size threshold of 2 on linear discriminant analysis (LDA) through the web site, http://huttenhower.sph.harvard.edu/galaxy [19, 21].

All data were expressed as means ± standard deviation. If some parameters were not normally distributed, nonparametric analysis was used. Results were compared between groups depending on the type of data analyzed using either the chi-square, Fisher's Exact, Student's t, or nonparametric Mann-Whitney U tests when appropriate. All statistical analyses were performed using Sample Power release 2.0 and SPSS for Windows version 14.0 (both by SPSS Inc, Chicago, IL, USA). All P values are two-tailed, and a P value l<0.05 was considered statistically significant.

## Results

### Metabolic effect in patients post subtotal gastrectomy

Compared with the control group, patients who had undergone subtotal gastrectomy (BII or RYGJ) had lower BMI, decreased waist circumferences, higher serum HDL, lower total cholesterol levels, lower serum TGs, lower serum glucose, and lower type II DM and MS occurrences (*P* < 0.05) (Table 1). There were no statistically significant differences in systolic or diastolic BPs between the two groups (*P* > 0.05) (Table 1).

**Table 1:** Anthropometric and laboratory data between patients with subtotal gastrectomy (Billroth II anastomosis and Roux-en Y gastrojejunal anastomosis) and patients without gastric surgery with a median follow-up of 8.25 years.

	Patients with subtotal gastrectomy (n = 111)	Control subjects (n = 344)	*P* value
**Age y/o**	69.4 ± 10.2	69.3 ± 10.5	0.565
**Sex (M: F)**	63: 48	199: 145	0.840
**body mass index**	21.6 ± 3.3	24.4 ± 3.0	<0.001
**waist (cm)**	77.4 ± 11.0	87.2 ± 8.7	<0.001
**systolic BP (mm Hg)**	123 ± 19	125 ± 18	0.338
**diastolic BP (mm Hg)**	74 ± 13	75 ± 10	0.338
**HDL-cholesterol (mg/dL)**	54 ± 12	50 ± 13	0.049
**Total cholesterol (mg/dL)**	163 ± 28	204 ± 39	<0.001
**Triglyceride (mg/dL)**	81 ± 33	118 ± 56	<0.001
**Serum glucose (mg/dL)**	100 ± 16	112 ± 22	0.049
**Diabetes (+/-)**	9: 102	56: 288	0.032
**Metabolic syndrome (+/-)**	14: 97	101: 243	<0.001

BP, blood pressure; HDL, high-density lipoprotein.

In subgroups analysis between subtotal gastrectomy patients with BII and control group, patients who had undergone subtotal gastrectomy with BII had lower BMI, decreased waist circumference, higher serum HDL, lower total cholesterol levels, and lower serum TGs than those of the controls (Table 2). However, there were no significant differences in systolic or diastolic BPs, serum glucose levels, and type II DM and MS occurrences between the two groups (*P* > 0.05) (Table 2). When compared with the controls, patients who had undergone subtotal gastrectomy with RYGJ had lower BMI, decreased waist circumference, lower serum glucose levels, lower total cholesterol levels, lower serum TGs, and lower DM and MS occurrences (*P* < 0.05) (Table 3). There were no significant differences in the systolic or diastolic BPs, and serum HDL between the two groups (*P* > 0.05) (Table 2).

**Table 2:** Anthropometric and laboratory data between patients with subtotal gastrectomy and patients without gastric surgery with a median follow-up of 8.25 years.

	Control subjects (n = 344)	Patients with B-II (n = 37)	Patients with RYGJ, (n = 74)
**Age y/o**	69.3 ± 10.5	69.9 ± 10.1	69.8 ± 11
**Sex (M: F)**	199: 145	23: 14	40: 34
**body mass index**	24.4 ± 3.0	21.6 ± 3.6 *	21.6 ± 3.2 ^#^
**waist (cm)**	87.2 ± 8.7	75.8 ± 10.9 *	78.5 ± 11.3 ^#^
**systolic BP (mm Hg)**	125 ± 18	122 ± 22	123 ± 18
**diastolic BP (mm Hg)**	75 ± 10	73 ± 14	74 ± 12
**HDL-cholesterol (mg/dL)**	50 ± 13	63 ± 15 *	60 ± 7
**Total cholesterol (mg/dL)**	204 ± 39	175 ± 30 *	158 ± 26 ^#^
**Triglyceride (mg/dL)**	118 ± 56	75 ± 26 *	84 ± 36 ^#^
**Serum glucose (mg/dL)**	112 ± 22	104± 19	98 ± 21 ^#^
**Diabetes (+/-)**	56: 288	4: 33	5: 69^#^
**Metabolic syndrome (+/-)**	101: 243	6: 31	8: 66 ^#^

BP, blood pressure; HDL, high density lipoprotein; B-II, subtotal gastrectomy + Billroth II anastomosis.

* P<0.05 when compared with the control group.

# P<0.05 when compared with the control group.

**Table 3:** Anthropometric and laboratory data between patients (subtotal gastrectomy) and controls with fecal microbiome with a median follow-up of 8.25 years.

	Patients with subtotal gastrectomy (n = 28)	Control subjects (n = 14)	*P* value
**Age y/o**	68.3 ± 10.2	68.5 ± 10.7	0.654
**Sex (M: F)**	12: 16	6: 8	1.000
**body mass index**	21.5 ± 3.2	23.3 ± 3.2	0.111
**waist (cm)**	76.8 ± 10.9	83.3 ± 11.8	0.095
**systolic BP (mm Hg)**	123 ± 22	122 ± 17	0.877
**diastolic BP (mm Hg)**	76 ± 13	77 ± 11	0.847
**HDL-cholesterol (mg/dL)**	56 ± 14	57 ± 15	0.961
**Total cholesterol (mg/dL)**	180 ± 29	206 ± 36	0.023*
**Triglyceride (mg/dL)**	83 ± 27	119 ± 44	0.012*
**Serum glucose (mg/dL)**	95 ± 9	100 ± 21	0.453
**Diabetes (+/-)**	0: 28	2: 12	0.106
**Metabolic syndrome (+/-)**	4: 24	5: 9	0.117

BP, blood pressure; HDL, high-density lipoprotein.

* P<0.05 when compared with the control group.

To investigate the long-term effects of subtotal gastrectomy on the gut microbiota, we further analyzed anthropometric, laboratory, and fecal microbiome from 14 patients who had undergone subtotal gastrectomy with BII, 14 study patients with RYGJ, and 14 controls of the same study subjects. Compared with the control group, patients who had undergone subtotal gastrectomy had significantly lower serum total cholesterol levels and TG (*P* < 0.05); and a trend to have lower BMI, decreased waist circumferences, lower occurrence of type II DM and MS (*P* all < 0.15) (Table 3).

### Statistical summaries of sequencing results

After 16S rRNA gene sequencing and quality filtering, 5.2 million reads from a total of 6.5 million pair-end reads were obtained corresponding to a mean of 83 ± 21 thousand reads per sample.

### Richness and diversity of gut microbiota

**Fig 1:**
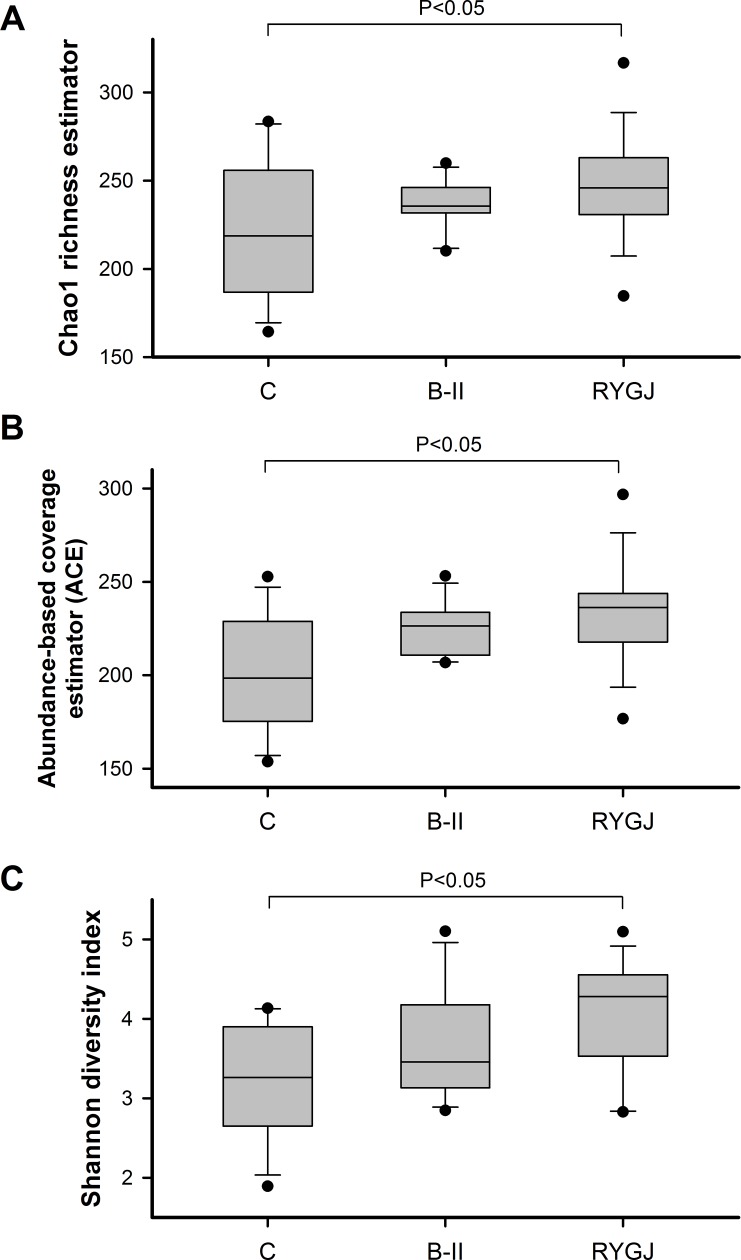
Richness and diversity of gut microbiota in the BII, RYGJ, and control groups. Chao1 and ACE indexes showed no significant difference in bacterial richness among B-II group and control group (*P* > 0.05). Chao1 and ACE indices showed significant differences in bacterial richness among RYGJ group and control group (*P* < 0.05). RYGJ group showed much higher bacterial diversity, as estimated by the Shannon diversity index (SI), in comparison with control group (*P* < 0.05). There was no significant difference in bacterial diversity among the BII and control groups (*P* > 0.05). The boxes (containing 50% of all values) show the median (horizontal line across the middle of the box) and the interquartile range, whereas the whiskers represent the 25th and the 75th percentiles. ACE, abundance‐based coverage estimators; C, control; B-II, subtotal gastrectomy with Billroth II anastomosis; RYGJ, subtotal gastrectomy with Roux-en-Y gastrojejunal anastomosis.

The gut microbiota richness was estimated by Chao1 and ACE (Fig 1A and 1B). Compared with the control group, the richness at the genera level in the BII group showed a tendency to increase (Chao, *P* = 0.08; ACE, *P* = 0.09). The RYGJ group showed significantly higher bacterial richness, in comparison with control group (Chao, *P* = 0.009; ACE, *P* = 0.0019).

The Shannon index (SI) was estimated to evaluate the ecological diversity of microbiota from each sample (Fig 1C). Compared with control group, the Shannon index at the genera level of BII group showed no difference (*P* = 0.185). The RYGJ group showed significantly higher bacterial diversity, in comparison with control group (*P* = 0.015).

### Long-term effects on gut microbiota composition

**Fig 2:**
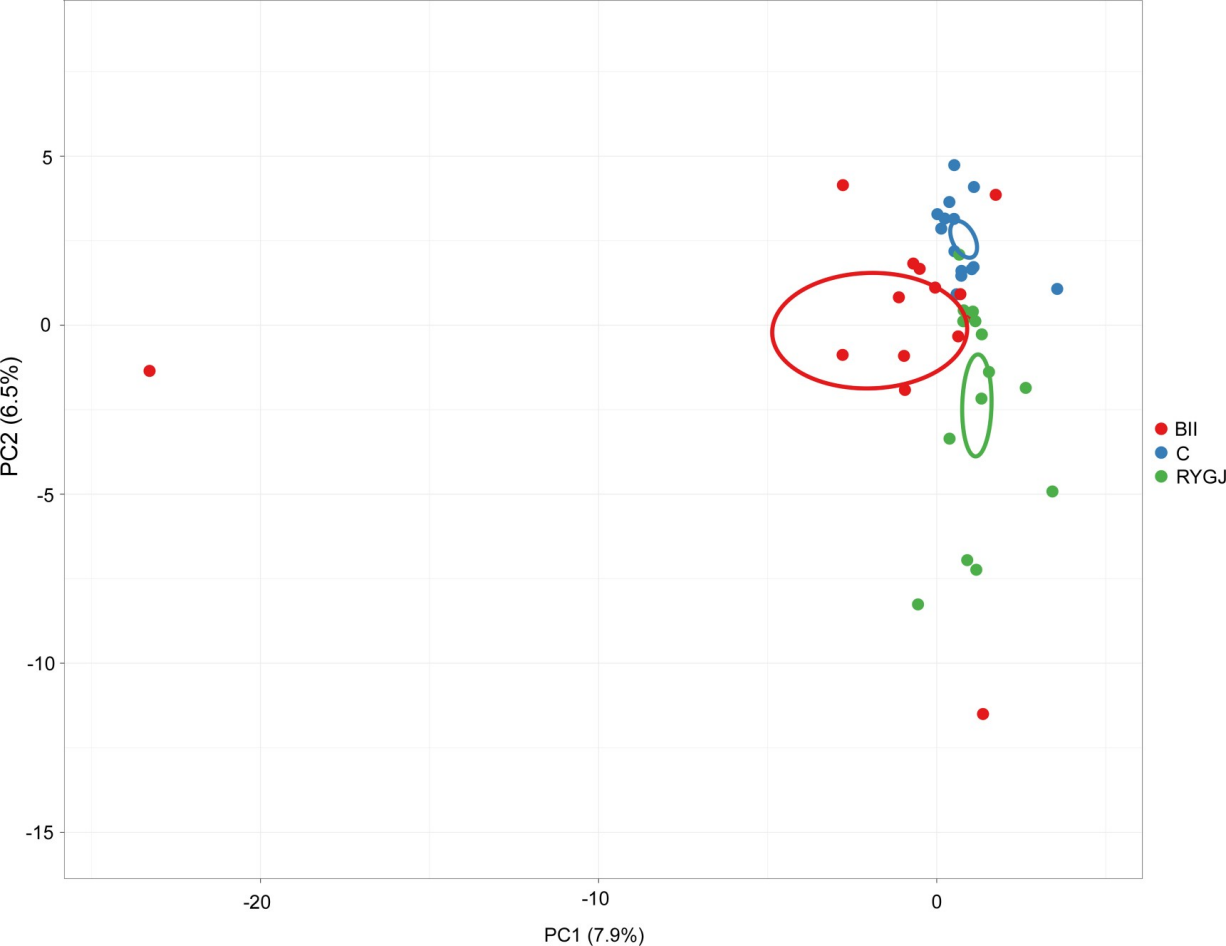
Principal component analysis of bacterial genera abundance. Principal component analysis of bacterial genera abundance showed a clear separation between control and BII or RYGJ groups. C, control; BII, subtotal gastrectomy with Billroth II anastomosis; RYGJ, subtotal gastrectomy with Roux-en-Y gastrojejunal anastomosis.

We visualized the changes in overall gut microbial genera composition using a PCA of the log-transformed relative abundances (Fig 2), which showed a clear separation between control group samples and those after BII or RYGJ. Compared with control group, PCA revealed significant differences in bacterial genera abundance after subtotal gastrectomy with BII (*P* < 0.001, Monte-Carlo simulation; Fig 2) and RYGJ (*P* < 0.001, Monte-Carlo simulation; Fig 2).

*Bacteroides*, *Firmicutes*, *Actinobacteria*, *Proteobacteria*, *Verrucomicrobia*, and *Fusobacteria* were the most abundant phyla in all three groups, which showed no differences in composition of above six phyla either between RYGJ and control groups or BII and control groups.

**Fig 3:**
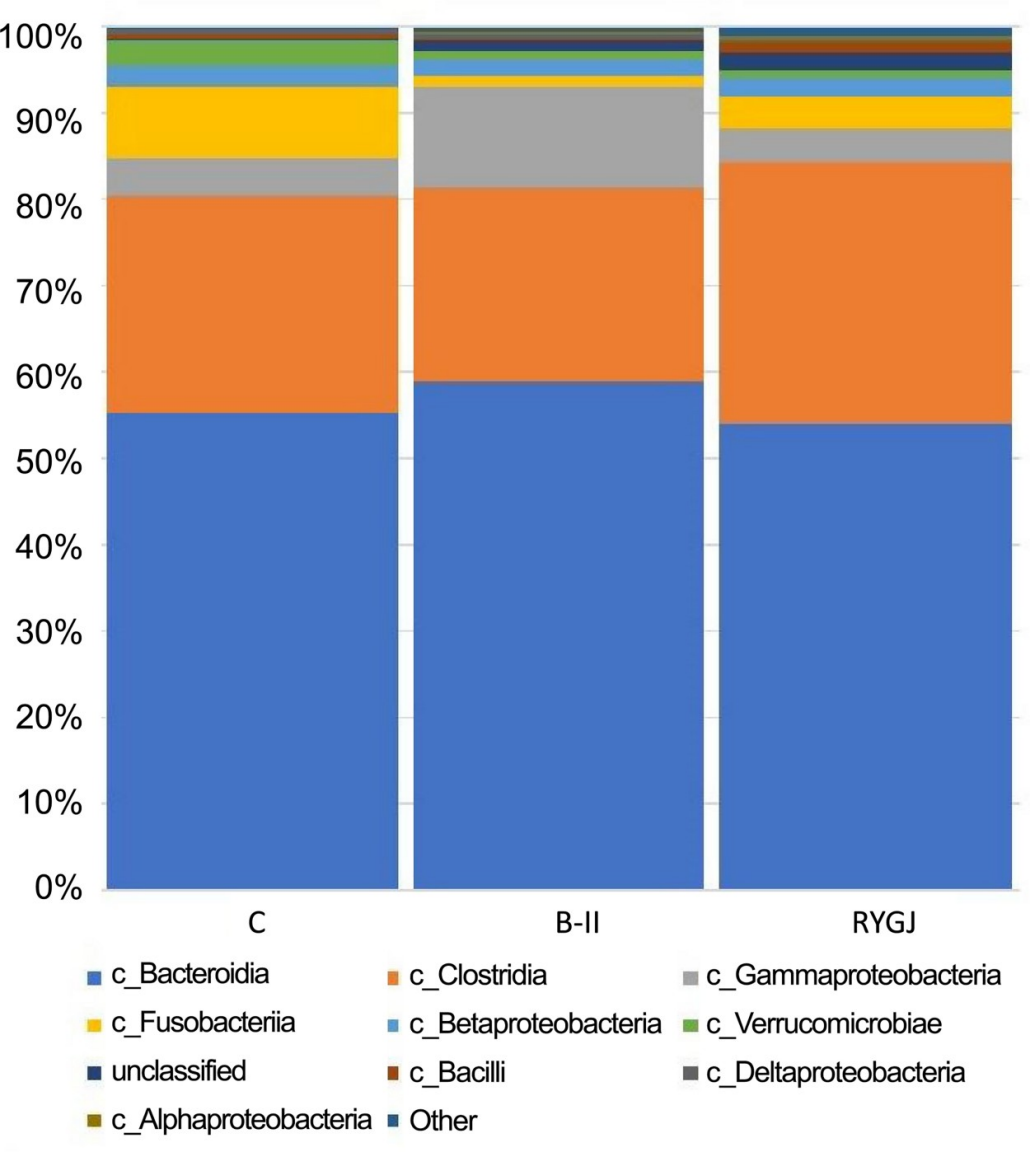
Relative abundances of classes across three groups. C, control; B-II, subtotal gastrectomy with Billroth II anastomosis; RYGJ, subtotal gastrectomy with Roux-en-Y gastrojejunal anastomosis.

The dominant class represented ≥ 0.2% of the resulting gut microbiota sequences. There were nine known dominant classes including *Bacteroidia*, *Clostridia*, *Gammaproterobacteria*, *Fusobacteriia*, *Betaproteobacteria*, *Verrucomicrobiae*, *Bacilli*, *Deltaproteobacteria*, and *Alphaproteobacteria* (Fig 3).

The bacterial class *Bacilli*, belonging to *Firmicutes*, was significantly higher in the RYGJ group compared with control group (1% versus 0.45%, *P* < 0.05). Moreover, compared with control group, the bacterial class *Alphaproteobacteria*, belonging to *Proteobacteria*, was significantly higher in RYGJ group (0.46% versus 0.006%, *P* < 0.05) and BII group (0.11% versus 0.006%, *P* < 0.05).

**Fig 4:**
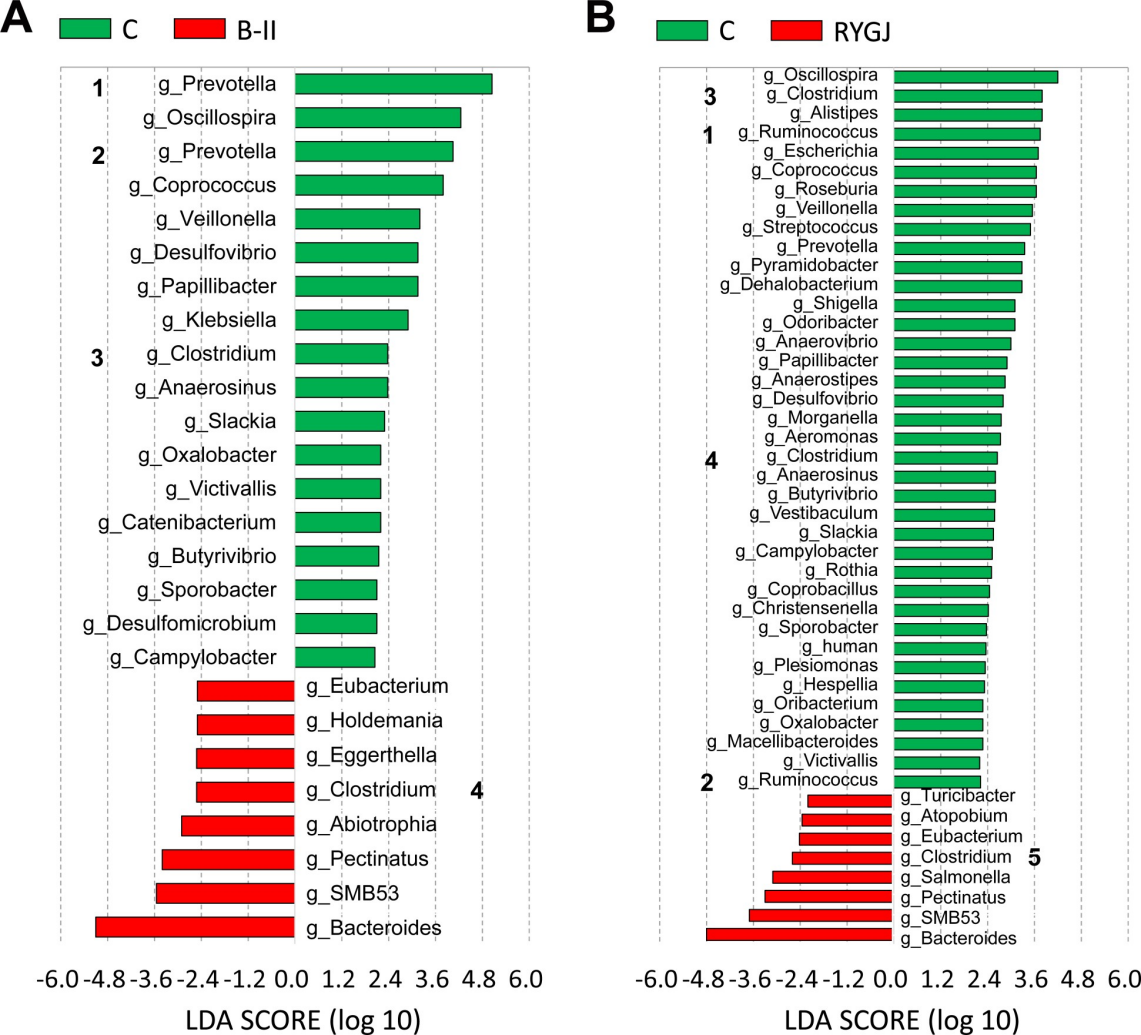
Known genera abundance reported by LEfSe in the bacterial community. Fig 4A. Known genera abundance reported by LEfSe in the bacterial community between B-II and control group. B-II, subtotal gastrectomy with Billroth II anastomosis.^1^This Prevotella genus is affiliated with Prevotellaceae. ^2^This *Prevotella* genus is affiliated with Paraprevotellaceae, a recommended family (based on the Greengenes database). ^3^This *Clostridium* genus is affiliated with *Ruminococcaceae*. ^4^This *Clostridium* genus is affiliated with *Peptostreptococcaceae*, a recommended family (based on the Greengenes database) Fig 4B. Known genera abundance reported by LEfSe in the bacterial community between RYGJ and control group. RYGJ, Roux-en-Y gastrojejuno anastomosis. ^1^This Ruminococcus genus is affiliated with Ruminococcaceae. ^2^This *Ruminococcus* genus is affiliated with Lachnospiraceae, a recommended family (based on the Greengenes database). ^3^ This *Clostridium* genus is affiliated with *Lachnospiraceae*. ^4^This *Clostridium* genus is affiliated with Ruminococcaceae. ^5^This *Clostridium* genus is affiliated with *Peptostreptococcaceae*, a recommended family (based on the Greengenes database).

Moreover, the linear discriminant analysis (LDA) effect size (LEfSe) analysis identified a total of 24 known genera, which were differentially abundant between the BII and control groups (Fig 4A). Among the 17 differentially abundant genera in BII group, *Prevotella* and *Oscillospira* represented the top two genera. There were a total of 43 known genera with differential abundance between RYGJ and control groups (Fig 4B). Among the 36 differentially abundant genera in the RYGJ group, *Oscillospira* and *Clostridium* represented the top two genera. Interestingly, *Oscillospira*, *Prevotella*, *Coprococcus*, *Veillonella*, *Clostridium*, *Desulfovibrio*, *Anaerosinus*, *Slackia*, *Oxalobacter*, *Victivallis*, *Butyrivibrio*, *Sporobacter*, and *Campylobacter* shared more abundant roles both in RYGJ and BII groups when compared with control group.

## Discussion

In our study, early gastric cancer patients who had undergone subtotal gastrectomy with B-II and RYGJ were found long-term benefits from the procedure on metabolic effects including lower BMI, decreased waist circumference, higher serum HDL, and lower serum TGs and glucose when compared with controls. Furthermore, patients who had undergone subtotal gastrectomy with RYGJ had significantly lower type II DM and MS occurrences when compared with those of the controls. Gut microbial richness and diversity also significantly increased after subtotal gastrectomy with RYGJ. Our data provided more medical information than previous studies in which improvement in DM status in patients who had undergone gastrectomy with RYGJ was reported [8, 9, 22, 23].

In the past, no studies had been conducted to explore the improvement in MS after curative gastric cancer surgery. In our study, patients in the RYGJ group had a significantly lower occurrence of MS than those of the controls. Moreover, patients in the BII group also had a trend of lower occurrence of MS than those of the controls. The aforementioned finding coincided with the finding that bariatric surgery produced a higher MS remission rate in patients with Roux-en-Y gastric bypass (RYGB) surgery than in patients who had undergone sleeve gastrectomy [10]. In addition to the foregut theory [7], the possible mechanisms for the differences in these procedures include: 1.) decrease in caloric intake, consequent weight loss, and reduction of fat mass; 2.) postoperative hormonal change in the enteroinsular axis including gastric inhibitory pepetide (GIP), glucagon-like peptide 1(GLP-1), peptide YY (PYY), and ghrelin [24]; 3.) modulation of intestinal nutrient sensing and regulation of insulin sensitivity [25]; and 4.) bile acid perturbations [25, 26].

Regarding the role of gut microbiota in regulation of the host metabolism this is the first study in which deep sequencing was applied to investigate gut microbiota in early gastric cancer patients who had undergone subtotal gastrectomy with BII and RYGJ. In parallel with metabolic improvement, gut microbial richness and diversity significantly increased after subtotal gastrectomy with RYGJ. Moreover, there was an increasing trend in the microbial richness after subtotal gastrectomy with BII. This result coincides with the results of the studies in gastric bypass bariatric surgery [14, 27]. One of the possible speculations to explain the increased gut microbial richness and diversity is the modulation of nutrient transit and physiology because subtotal gastrectomy with RYGJ and BII drastically modify the anatomy of the gastrointestinal system. Moreover, it is reported that RYGB induces a drastic reduction in food intake with modifications in the nutrient supply, such as an increase in polysaccharide consumption and a reduction in fat consumption [28]. In countries where people decrease their fat intake and increase polysaccharides consumption, the overall diversity of gut microbiota increased when compared with that of consumers of Western diets [29].

In our study, although there were no differences in the phyla of the gut microbiota among the three study groups, and we found an obvious shift of the gut microbiota toward a higher abundance of *Alphaproteobacteria* (a main branch of *Proteobacteria*) and *Bacilli* at the class level in subjects with subtotal gastrectomy with RYGJ. Moreover, an increase in *Alphaproteobacteria* (at the class level) was also found in subjects after subtotal gastrectomy with BII. These data supported the concept that *Alphaproteobacteria* might be associated with the beneficial regulation of glucose metabolism in post-gastric surgery patients [30]. However, other studies involving human subjects and rats showed a proportional increase in *Gammaproteobacteria*, also a main branch of *Proteobacteria*, in subjects who had undergone with gastric (bariatric) surgery [13, 31, 32]. Inconsistent results between these studies and ours may be related to different indications and gastric surgery modalities for different underlying diseases. One speculation is the original microbiota status of obesity subjects may be different from the original microbiota status of gastric cancer patients. Therefore, it is reasonable the subsequent microbiota status may be different in gastric cancer patients after subtotal gastrectomy and obesity patients after bariatric surgery.

In agreement with previous studies, an important increase in aero-tolerant *Proteobacteria*, including *Streptococcus* and *Escherichia,* in patients who had undergone subtotal gastrectomy with RYGJ, and *Klebsiella* in patients who had undergone subtotal gastrectomy with BII was observed in our study. The change of gut microbiota after subtotal gastrectomy may be a result of anatomical rearrangements contributing to higher presence of oxygen in distal parts of the gut [12–14, 33, 34]. Possible mechanisms common to the two surgical procedures (BII and RYGJ) might affect the composition of the gut microbiota. First, a decrease in acid secretions has been shown to promote the growth of *E*. *coli in vitro*, and both subtotal gastrectomy with RYGJ and BII are associated with reduced luminal exposure to gastric acid [35]. Second, increases in pH due to the reduced size of the stomach after subtotal gastrectomy could make the gastric barrier less stringent for oral microbiota such as *Streptococcus* spp., together with a few *Veillonella* spp., which are metabolically dependent on *Streptococcus* spp. in oral biofilms [36].

Taken together, compared with previous studies which focused on the impact of bariatric surgery on gut microbiota, our study has reproduced previously observed changes in increasing some genera (*Veillonella, Klebsiella, Alistipes*, *Roseburia*, *Streptococcus*, *Shigella*) in patients after subtotal gastrectomy with BII and RYGJ although the purpose of the surgery between the two population is completely different [12,14,27, 33, 34]. Moreover, the two surgical procedures, including subtotal gastrectomy with BII and RYGJ, produced similar and durable changes in gut microbiome which showed more abundant genera including *Oscillospira*, *Prevotella*, *Coprococcus*, *Veillonella*, *Clostridium*, *Desulfovibrio*, *Anaerosinus*, *Slackia*, *Oxalobacter*, *Victivallis*, *Butyrivibrio*, *Sporobacter*, and *Campylobacter* possibly because of the similarities between the two surgical procedures in the GI tract.

Our study showed that patients with early gastric cancer who had undergone subtotal gastrectomy (BII or RYGJ) had subsequent lower BMI and waist circumference, but only patients with RYGJ had subsequent lower occurrence of type II DM and MS when compared to that of the controls. According to these findings, it was recommended that patients with MS and early gastric cancer should receive subtotal gastrectomy with RYGJ rather than another surgical procedure in order to improve their metabolic conditions. Although the interactions between subtotal gastrectomy, metabolic improvement, metabolomics, and gut microbiota are not well understood, the possible potential crosstalk might include: 1.) a decrease in circulating levels of lipopolysaccharides and altered bacterial components known to promote hepatic insulin sensitivity [37,38]; 2.) the impact of gut microbiota on the host’s enteroendocrine secretion [39, 40]; and 3.) alteration in bile acid flow, which is certainly a driver for the changes in microbial population [41]. Some randomized clinical studies may be needed to clarify the metabolic effects and possible mechanisms involved in microbiota, functional metagenomics, and metabolomics in this interesting issue.

Our study has a number of strengths. First, we observed long-term (at least eight years) effects of metabolic profiles and microbiota statuses in early gastric cancer patients after curative surgery and subtotal gastrectomy with BII or RYGJ. Second, a medium sample size for the metabolic analysis and suitable sample size for the analysis of microbiome was obtained. This study has several limitations that are worth noting. First, this study was a cross-sectional study lacking a prospective longitudinal long-term follow-up in the evolution of MS and gut microbiota. Second, this study has a lack of data about gut hormones such as GIP, GLP-1, PYY, and ghrelin, all of which also play an important role in the change of diabetic status and MS in patients who have undergone bariatric surgery. Third, we acknowledge that an important drawback of our study is the relatively small number of patients. Fourth, we did not record antibiotic exposures during the follow-up years, we cannot know if there were differences in antibiotic exposures between our study groups. It is known that antibiotics alter gut microbiota, most constituents of the gut microbiota return to preantibiotic levels within 4 weeks although some species may fail to recover to preantibiotic levels for much longer periods after the end of antibiotic therapy [42, 43]. Fifth, we did not have metatranscriptomic, proteomic, and metabolomic data to demonstrate causal relationship. Finally, we did not find out and correlate the association between individual gut microbiota and individual parameters of the metabolic syndrome in patients after subtotal gastrectomy with RYGJ and BII. We just found the association between subtotal gastrectomy, change of gut microbiota and metabolic prolife rather the causal effect between them.

## Conclusions

Early gastric cancer patients who had undergone subtotal gastrectomy with RYGJ had a lower occurrence of MS and type II DM than the controls during long-term (> 8-year) follow-up periods. In parallel with metabolic improvements, gut microbial richness and diversity also increased significantly after subtotal gastrectomy with RYGJ. Further studies are needed to clarify the metabolic effects and possible mechanisms involving in microbiota, functional metagenomics, and metabolomics in this interesting issue.
